# Editorial: Sustainable Development Goals (SDGs): Impact on Nutrition

**DOI:** 10.3389/fnut.2021.676080

**Published:** 2021-05-28

**Authors:** Kathleen L. Hefferon, Shauna Downs, Gemma Oms Oliu, Hans De Steur

**Affiliations:** ^1^Department of Microbiology, Cornell University, Ithaca, NY, United States; ^2^Department of Urban-Global Public Health, Rutgers University, Newark, NJ, United States; ^3^Department of Food Technology, Universitat de Lleida, Lleida, Spain; ^4^Department of Agricultural Economics, Ghent University, Ghent, Belgium

**Keywords:** sustainable development goals, nutrition, policy, consumer behavior, global research

The seventeen Sustainable Development Goals (SDGs), set in place by the United Nations General Assembly in 2015, provides a “blueprint to achieve a better and more sustainable future for all” by 2030. The SDGs range from reducing global poverty and hunger, to tackling climate change and preserving the environment. The SDGs are interconnected and synergistic by nature—actions toward addressing one goal will likely affect the progress toward achieving many more. For example, healthy diets and good nutrition, which are encompassed in SDG 2 (“no hunger”) and SDG 3 (“good health and well-being”), have the potential to impact a myriad of other goals related to productivity, education, equity, and beyond. By improving diets and nutrition, the world will be better positioned to achieve the SDGs by 2030. The subject of nutrition is overly complex and requires an examination of the intricate relationships between nutrition and the SDGs through a variety of different lenses. It seems timely and appropriate, therefore, to review and critically analyze the SDGs and their targets as they pertain to the field of nutrition.

This Research Topic consists of five contributions, with themes ranging from marketing and consumer behavior to food nutrition and health policy. Within each of the studies included within this Research Topic, a final section links its results and conclusions to the SDGs. The papers discussed in this Research Topic cover developing and developed regions, from Vietnam to Italy, from India to sub-Saharan Africa. Much of the studies deal with the nutritional content of food, consumer behavior, and the current policy structures which influence them. The North-South challenge of the SDGs are intertwined with the broad geographic scope of the articles presented. These topics directly address SDGs 1 and 2, No Poverty and Zero Hunger, as well as 3, Good Health and Wellbeing. However, many of the papers included in this topic have the potential to reach beyond SDGs 1–3 through more indirect pathways. For example, in “*Supermarkets and Household Food Acquisition Patterns in Vietnam in Relation to Population Demographics and Socioeconomic Strata: Insights From Public Data*” by Trinh et al., the rapidly changing food environment in fast-paced Southeast Asia is examined. As the traditional market in Vietnam is replaced by an expansion of supermarkets, food quality and quantity acquired by households are undergoing transformations. The authors were able to record differences in household food acquisition and food diversity depending on the number of nearby supermarkets, wealth, ethnicity, and household size. The data collected from this and similar studies can be used to inform policy recommendations regarding food policy and consumer behavior.

The research article by Ferrari et al., “*Could Dietary Goals and Climate Change Mitigation Be Achieved Through Optimized Diet? The Experience of Modeling the National Food Consumption Data in Italy*” sought to use survey data to design a healthy diet model for Italy that also produced low greenhouse gas emissions, thus directly addressing SDGs 2, 3, and 12. In “*Leveraging Traditional Ecological Knowledge and Access to Nutrient-Rich Indigenous Foods to Help Achieve SDG 2: An Analysis of the Indigenous Foods of Sauria Paharias, a Vulnerable Tribal Community in Jharkhand, India*” Ghosh-Jerath et al. explore the indigenous food system of the Sauria Paharia tribe in rural India and how it could be leveraged to improve diets and reduce malnutrition. The authors examined the nutritional composition of indigenous foods and consumption patterns among villagers and discussed policies that could help promote nutrient rich indigenous foods as part of diet diversification strategies.

Consumer purchasing behavior in sub-Saharan Africa was also a theme in this Research Topic. In “*Labeling Nutrition-Sensitive Food Chains: A Consumer Preference Analysis of Milk Products*” by Wesana et al., the Ugandan dairy sector was examined as a case study in exploring the potential of using a nutrition-sensitive chain label in providing more positive perceptions for consumers when aiming to purchase dairy products. This work can be used to develop policy surrounding labeling interventions in the future. Similarly, the study by Oteh et al., “*Moving Biofortified Cassava Products Closer to Market in Nigeria*” sought to examine consumer purchasing behavior with respect to biofortified cassava. The study found that consumers do not understand the improved nutritional value of biofortified cassava and identified where policy interventions could increase public awareness and demand for this crop.

The aim of this Research Topic was to provide examples of the ways in which actions to improve diets and nutrition have the potential to impact SDGs globally. This collection of research articles has revealed how emerging academic research in nutrition can assist in directing future policies that would facilitate the achievement of these nutrition-oriented goals within a timeframe that is both realistic and in concert with the other SDGs. These articles also elicit recommendations for future research and practice concerning the areas of study presented in this Research Topic.

## Author Contributions

All authors listed have made a substantial, direct and intellectual contribution to the work, and approved it for publication.

## Conflict of Interest

The authors declare that the research was conducted in the absence of any commercial or financial relationships that could be construed as a potential conflict of interest.

